# The Expression of Semaphorin3E in Vagal Ganglion and Lung Tissue Is Related to Airway Hyperresponsiveness in Murine Asthma Model

**DOI:** 10.1155/2023/6459234

**Published:** 2023-12-11

**Authors:** Liyan Chen, Xiaohui Yuan, Yaowei He, Zichuan Fan, Ya Guan, Qiuying Li, Yaying Chen, Lianglan Bao, Yidan Huang, Kefang Lai

**Affiliations:** ^1^The First Affiliated Hospital of Guangzhou Medical University, National Center of Respiratory Medicine, State Key Laboratory of Respiratory Disease, Guangzhou Institute of Respiratory Health, Guangzhou 510120, Guangdong, China; ^2^Shenzhen Hyzen Hospital, Shenzhen 518000, Guangdong, China; ^3^Guangdong Second Provincial General Hospital, Guangzhou 510317, Guangdong, China; ^4^The Affiliated Dongguan Houjie Hospital of Guangdong Medical University, Dongguan 523945, Guangdong, China

## Abstract

**Objective:**

Semaphorin3E (Sema3E) mediates reorganization of the actin cytoskeleton, and plays an important role in ensuring the specificity of synapse formation and angiogenesis. However, the role of Sema3E in allergic asthma (AS) and eosinophilic bronchitis (EB) is still elusive. This study aimed to investigate the relationship between Sema3E in vagal ganglion and lung tissue, airway reactivity, and eosinophilic inflammation.

**Methods:**

The frequency of coughs and airway reactivity as well as the airway inflammation were observed in ovalbumin- (OVA-) induced AS and EB mouse models. The expression of Sema3E was examined in the vagal ganglion and lung tissues by immunofluorescence staining and western blotting analyses. In the Sema3E treatment protocol, exogenous Sema3E was administrated intranasally before challenge in AS model to study the effect of Sema3E on airway hyperresponsiveness, airway inflammation, mucus production, and collagen deposition.

**Results:**

The similar higher frequency of coughs and airway eosinophilic inflammation could be seen in AS and EB groups compared with nasal saline (NS) and dexamethasone (DXM) groups. The absence of the airway hyperresponsiveness was observed in EB and DXM group, while AS group showed increase in airway reactivity to methacholine. The expression of Sema3E in vagal ganglion and lung tissue was remarkably decreased in AS and DXM group compared with EB group. Sema3E-treated asthma mice displayed ameliorated airway hyperresponsiveness, mucus production, and collagen deposition.

**Conclusion:**

Sema3E in lungs and vagal ganglia is related to eosinophilic inflammation and has a protective effect on OVA-induced AHR in asthma.

## 1. Introduction

Allergic asthma is a common chronic lung disease in the world, characterized by reversible airflow limitation, expiratory dyspnea, and wheezing [1]. Airway hyperresponsiveness (AHR) is an important pathophysiological feature of asthma, which means excessive bronchoconstriction in response to internal and external stimulus, thereby causing symptoms such as wheezing [2, 3]. The mechanism of AHR is very complex, which is not yet fully understood. It has been confirmed to be related to many factors, such as airway inflammation, oxidative stress, airway remodeling, and neurological dysfunction [4, 5]. Therefore, it is very important for us to find a key biomarker of AHR. Interestingly, we found that asthma performed similar clinical manifestations to another respiratory disease, namely eosinophilic bronchitis (EB), which was one of the common causes of chronic cough, characterized by cough, airway eosinophilic inflammation, responsive to steroid therapy, while absence of airway responsiveness [6]. Long-term follow-up studies have shown that EB was an independent disease rather than the early stage of asthma or chronic obstructive pulmonary disease [7]. Furthermore, EB and asthma perform similar airway inflammation but different airway responsiveness, which makes the EB model a good control group for studying the mechanism of airway hyperresponsiveness in the bronchial asthma. In previous studies, we have successfully established an EB mouse model which was conducive to explore the mechanism of AHR in allergic asthma [8].

Semaphorins, a large family of multifunctional media found in the different organs, originally discovered as guidance cues for developing axons, play an important role in the nervous system, cardiovascular system, immune system, and respiratory system, and participate in the regulation of angiogenesis, embryonic development, airway disease, and tumor formation [9]. Semaphorin3E (Sema3E), an important member of the Semaphorins family, binds to the receptor PlexinD1 and then plays an important role in the axon guidance, immune regulation, cell migration, proliferation, and angiogenesis [10, 11]. Studies have shown that downregulation of Sema3E could promote the proliferation and contraction of smooth muscle cells in the airway, increase airway collagen deposition and promote mucus production, which ultimately led to AHR in dust mites sensitized Sema3E-/- asthma model, but after Sema3E treatment, the airway responsiveness was significantly reduced [12]. In asthma models sensitized by other substances (such as ovalbumin and diesel exhaust), whether Sema3E can effectively reduce airway responsiveness is unknown. The sensory nerve endings that innervate the airway smooth muscle come from the nerve fibers sent by the vagal nerve. Studies have shown that ozone-induced AHR was caused by substance P released by parasympathetic neurons of the airway ganglion. Substance P could increase airway smooth muscle contraction, and indirectly enhanced cholinergic nerves to release peripheral acetylcholine that caused excessive bronchial constriction [13, 14]. Besides, MrgprC11 expressed in the vagal ganglion could enhance airway contraction and led to AHR [15], suggesting that the vagal nerve played an important role in the occurrence of asthma AHR. However, whether Sema3E in the vagal ganglion can regulate AHR has not been reported yet. Therefore, this study aimed to observe the Sema3E levels expressed in the vagal ganglion and lung tissues in OVA-induced asthma and EB mouse models, and to investigate the possible therapeutic effects of Sema3E on asthma.

## 2. Materials and Methods

### 2.1. Animals

BALB/c mice (7-week-old) were obtained from the central animal house of Southern Medical University (Guangzhou, China), and housed in the specific pathogen-free conditions in the State Key Laboratory of Respiratory Disease. All procedures were approved by the University of Guangzhou Medical University Animal Care and Ethics Committee.

### 2.2. Establishment of Mouse Models

Mouse models were established as previously described [8]. In brief, on Days 0, 7, and 14, the AS, EB, and DXM groups were administered with OVA (Sigma Company, USA, A5503-10G) sensitization solution at 200 *μ*L/animal (containing 10 *µ*g of OVA + 1.3 mg of aluminum hydroxide adjuvant (Thermo Fisher Scientific, USA, 77161)) by intraperitoneal injections and challenged with 200, 10, and 10 *µ*g OVA on Days 21–23, respectively. Additionally, the DXM group was treated with 5 mg/kg of DXM (Guangzhou Baiyunshan Tianxin Pharmaceutical Co., Ltd., China, H44022091) by intraperitoneal injection 1 hr before stimulation and measurement of airway reactivity. The NS group was administered equal amount of NS for intraperitoneal sensitization and intranasal stimulation.

**Figure 1:**
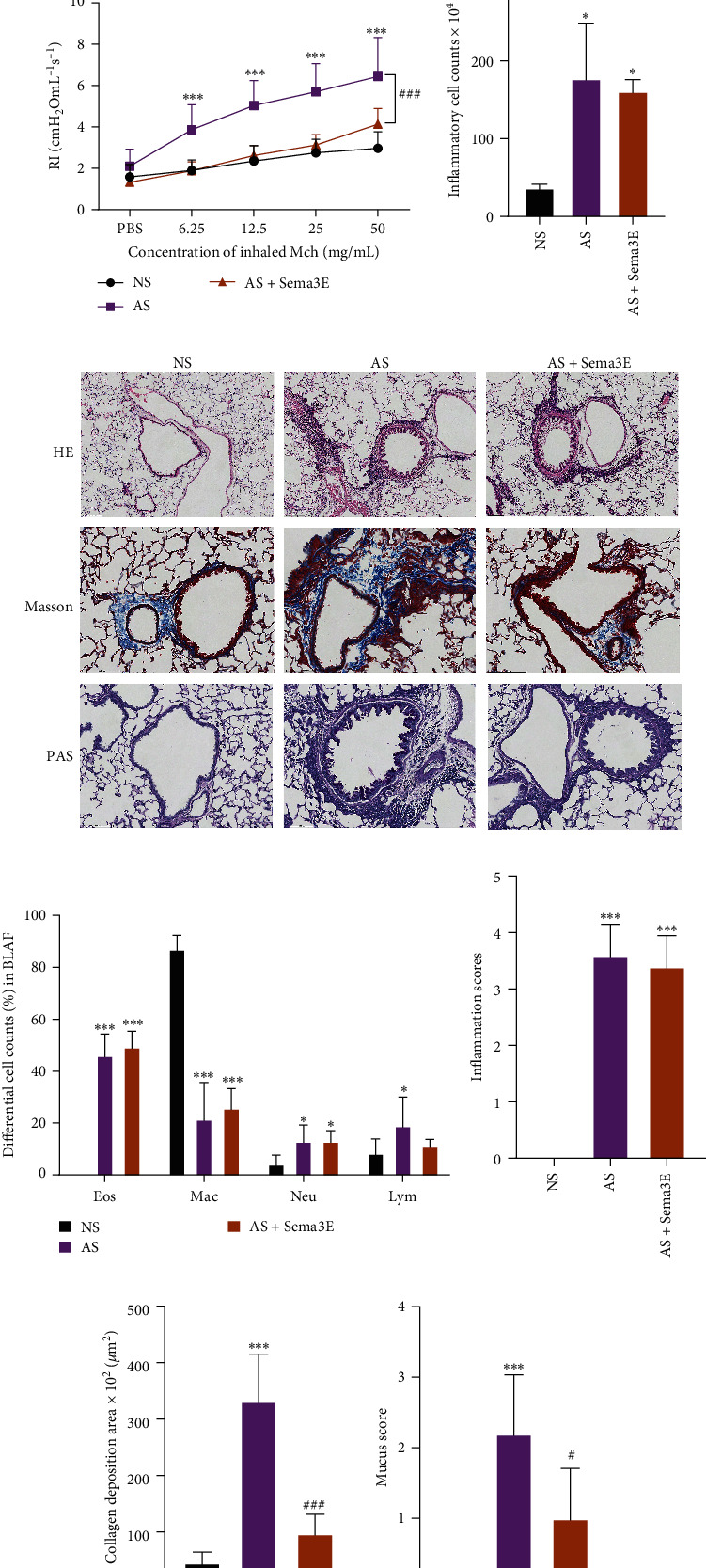
Sema3E ameliorated the airway hyperresponsiveness, collagen deposition and mucus production in AS model. (a) Protocol outline. Mice were sensitized intraperitoneally with 10 *µ*g OVA (or NS as control) on Days 0, 7, and 14. On Days 21–23, mice were challenged i.n. with 200 *µ*g OVA (or NS as control), mice were exposed intranasally to Sema3E 1 hr before challenge in AS + Sema3E group. (b) Lung resistance in response to inhaled methacholine 24 hr after the last OVA challenge. (c) The total inflammatory cell counts in the BALF of mice for all groups. (d) Representative lung sections stained with hematoxylin and eosin (HE), periodic acid–Schiff (PAS), and Masson staining. (e) Differential cell counts in BALF. (f) Scoring of leukocyte infiltration in HE-stained lung tissue sections. (g) Quantification of collagen deposition. (h) Quantification of mucus-producing goblet cells. Data in (b)–(h) were expressed as mean ± standard deviation (*n* = 5). Compared with the NS group,  ^*∗*^: *p* < 0.05;  ^*∗∗*^: *p* < 0.01,  ^*∗∗∗*^: *p* < 0.001, compared with AS group, ^#^: *p* < 0.05, ^##^: *p* < 0.01, ^###^: *p* < 0.001. HE staining: magnification = ×100, scale bar = 100 *µ*m, PAS and Masson staining: magnification = ×200, scale bar = 100 *µ*m. (NS: nasal saline group, AS: asthma group, EB: eosinophilic bronchitis group, DXM: dexamethasone group, Sema3E: Semaphorin3E, OVA: ovalbumin, MCh: methacholine, Eos: eosinophil, Neu: Neutrophil, Mac: macrophage, and Lym: lymphocyte).

In the Sema3E treatment protocol (Figure 1(a)), the AS mouse model was established as described above while challenged i.n. with OVA (200 *µ*g in 25 *µ*L NS), mice were exposed intranasally to Sema3E (5 *µ*g in 25 *µ*L NS) (R&D Systems, Minneapolis, MN., USA, 3239-S3B-025), or NS as a control 1 hr before challenge. Twenty-four hours after the last administration, mice were anesthetized for invasive airway resistance detection and then sacrificed for analysis of airway inflammation, mucus production, and collagen deposition.

### 2.3. Measurement of Cough Frequency

According to the mouse cough detection method established previously by our group [8], mice were placed in the Buxco noninvasive plethysmography chamber and then a mini-microphone was mounted onto the lateral aperture of the plethysmograph, which can connect the output port to the computer, and record the sound waves through Cooledit sound analysis software. 1-mL Capsaicin (0.1 mmol/L, Sigma Company, USA, 211275) was atomized for 3 min, then the number of mice coughs was recorded of total 6 min.

### 2.4. Detection of Airway Reactivity

The invasive airway resistance detection of mice was measured by Buxco's invasive airway resistance and lung compliance detection system as described previously [8]. In the Sema3E treatment protocol, mice were anesthetized for invasive airway resistance detection 24 hr after the last administration. Lung resistance was measured after 1x phosphate buffered saline (PBS, 0.01 M) stimulation and increasing concentration of methacholine (6.25–50 mg/mL) (MCh, Sigma Company, USA, A6625).

### 2.5. Classification of Cells in BALF

After detection of the airway reactivity, the mice were sacrificed for collection of BALF. Lungs received three sequential 0.8 mL lavages of ice cold 1x PBS delivered into the airways through a 1 mL sterile syringe in the mouse trachea. The bronchoalveolar lavage fluid (BALF) was centrifuged at 4°C, 1,500 rpm × 10 min, and the supernatant was stored at −80°C. The cell pellet was resuspended in 1x PBS, and 10 *µ*L resuspension was taken on a cell counting plate. The total number of white blood cells was counted under a microscope, and the rest of the cell suspension was coated on a glass slide for HE staining and Eos%, Neu%, Mac%, and Lym% were calculated.

### 2.6. Isolation of Vagal Ganglion

Under the microscope, the carotid sheath was founded along the side of the trachea, then the vagal nerve was gently peeled off, and the vagal ganglion was separated upward along the vagal nerve and placed in 10% neutral formaldehyde for external fixation (Jingxinbio, China, JX0300). Then dehydration, transparency, embedding, sectioning, rinsing with gradient alcohol, and double distilled water were performed. Fluorescent staining was performed afterward.

### 2.7. Lung Histology

The right lungs of the mice were obtained for paraffin section and fixed in 4% of paraformaldehyde solution (Meilunbio, China, MA0192) for more than 24 hr. The lung sections were stained with hematoxylin and eosin (HE), periodic acid–Schiff (PAS) staining and Masson staining according to the standard protocols. The extent of mucus production was quantified in a blind manner on PAS-stained lung sections by a score according to the percentage of goblet cells in the epithelial cells [16]: 0: no goblet cells, 1: <25%, 2: 25%–50%, 3: 50%–80%, and 4: >80%. The area of collagen deposition was quantified using the Image-Pro Plus software (Media Cybernetics, USA).

### 2.8. Expression of Sema3E in Vagal Ganglia and Lung TIssues

The frozen sections from vagal ganglia and lung tissues were examined by immunofluorescence staining to detect the expression of Sema3E, and incubated with Sema3E goat antibody (R&D Systems, USA, AF3239) overnight at 4°C. The tissues were incubated with the secondary antibody (Abcam, USA) for 1 hr at room temperature followed by washing for three times. Besides, the expression of Sema3E in lung tissues was examined by western blot. Protein samples were isolated from homogenized tissues and the total protein concentration was analyzed through BCA Protein Assay Kit (Thermo Fisher Scientific, USA, 23227). The proteins were isolated by 4%–20% sodium dodecyl sulfate–polyacrylamide gel electrophoresis (SDS–PAGE) (Genshare Biological, China, JC-PE022) and then transferred to PVDF membrane (Merck, USA, IPVH00010) at 250 mA for 100 min, which were blocked using the fast blocking liquid (Beyotime Biotechnology, China, P0231). The proteins were incubated with the following primary antibodies overnight at 4°C: Sema3E Rabbit Antibody (ABclonal, China, A18409), *β*-actin (Earthox, USA, E021020), and the secondary antibody that was antirabbit immunoglobulin G (Cell Signaling Technology, USA, 7074S) at room temperature for 1 hr. Electrochemiluminescence (Beyotime Biotechnology, China, P0018S) was used to detect the proteins.

## 3. Statistical Analysis

All data were expressed as mean ± standard deviation. One-way analysis of variance (ANOVA) was performed to analyze the differences among the groups. For the comparison of airway hyperresponsiveness, a two-way ANOVA with Tukey's post hoc test was used. All statistical analyses were performed using GraphPad Prism 8.0 and SPSS 25.0 software. Significance for all tests was assessed as *p* < 0.05.

## 4. Results

### 4.1. The AS and EB Models Showed Similar Higher Cough Frequency and Increased Lung Inflammation but Different Airway Reactivity

**Figure 2:**
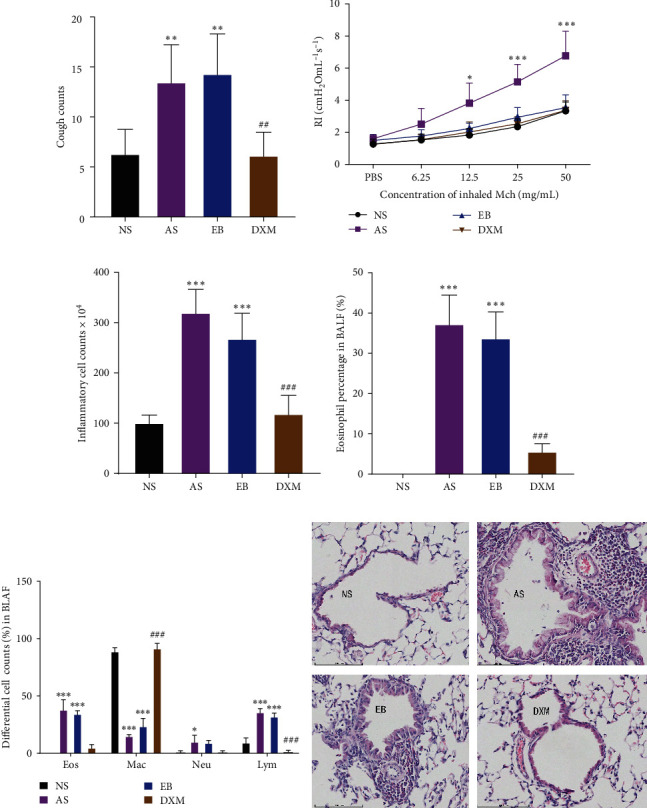
OVA-induced asthma and EB mouse models showed similar airway inflammation but different airway hyperresponsiveness. (a) The number of coughs in the four groups of mice. (b) The airway reactivity of mice in four groups. (c) The total inflammatory cell counts in the BALF of mice for the four groups. (d) Eos% in BALF of mice in the four groups. (e) Differential cell counts in BALF. (f) The lung tissue pathological sections of the four mice groups were stained with HE. (Compared with NS group,  ^*∗*^: *p* < 0.05;  ^*∗∗*^: *p* < 0.01,  ^*∗∗∗*^: *p* < 0.001; compared with EB group, ^#^: *p* < 0.05; ^##^: *p* < 0.01, ^###^: *p* < 0.001). Data were expressed as mean ± standard deviation (*n* = 6) (NS: nasal saline group, AS: asthma group, EB: eosinophilic bronchitis group, DXM: dexamethasone group, MCh: methacholine, Eos: eosinophil, Neu: Neutrophil, Mac: macrophage, and Lym: lymphocyte).

When AS and EB models were established, the number of coughs in the AS group (13.5 ± 3.7) and EB group (14.3 ± 4.0) was significantly increased than that of the NS group (6.3 ± 2.4). Compared with EB group, the number of coughs (6.2 ± 2.3) in the DXM group was reduced (*p* < 0.01) (Figure 2(a)). However, the lung resistance in the AS group after atomization of MCh (12.5, 25, and 50 mg/mL) was significantly higher than that in EB group, DXM group, and NS group (*p* < 0.05). There was no significant difference in RI value among above three groups (*p* > 0.05) at each concentration of MCh (6.25–50 mg/mL) after challenge (Figure 2(b)). What is more, the total number of white blood cells in the BALF of the AS group (320.4 ^*∗*^ 10^4^ ± 45.8 ^*∗*^ 10^4^) and EB group (268.7 ^*∗*^ 10^4^) ± 50.0 ^*∗*^ 10^4^) was significantly higher compared with the NS group (101.5 ^*∗*^ 10^4^ ± 14.8 ^*∗*^ 10^4^) and DXM group (119.2 ^*∗*^ 10^4^ ± 36.3 ^*∗*^ 10^4^) (Figure 2(c)). Eos% (37.3% ± 7.1%) in AS group and (33.8% ± 6.5%) in EB group were significantly higher than those in NS group (0%) and DXM group (5.7% ± 1.9%) (*p* < 0.001) (Figure 2(d)). The eosinophil and lymphocyte counts in the BALF in AS and EB groups were increased compared to the NS group. Compared with EB group, DXM administration could increase macrophage but decrease lymphocyte counts (Figure 2(e)). Compared with EB group, the inflammatory cells around the airway in AS group were more infiltrated while significantly decreased in DXM group (Figure 2(f)).

### 4.2. The Expression of Sema3E in the Vagal Ganglion and Lung TIssues Was Significantly Increased in EB Mice

**Figure 3:**
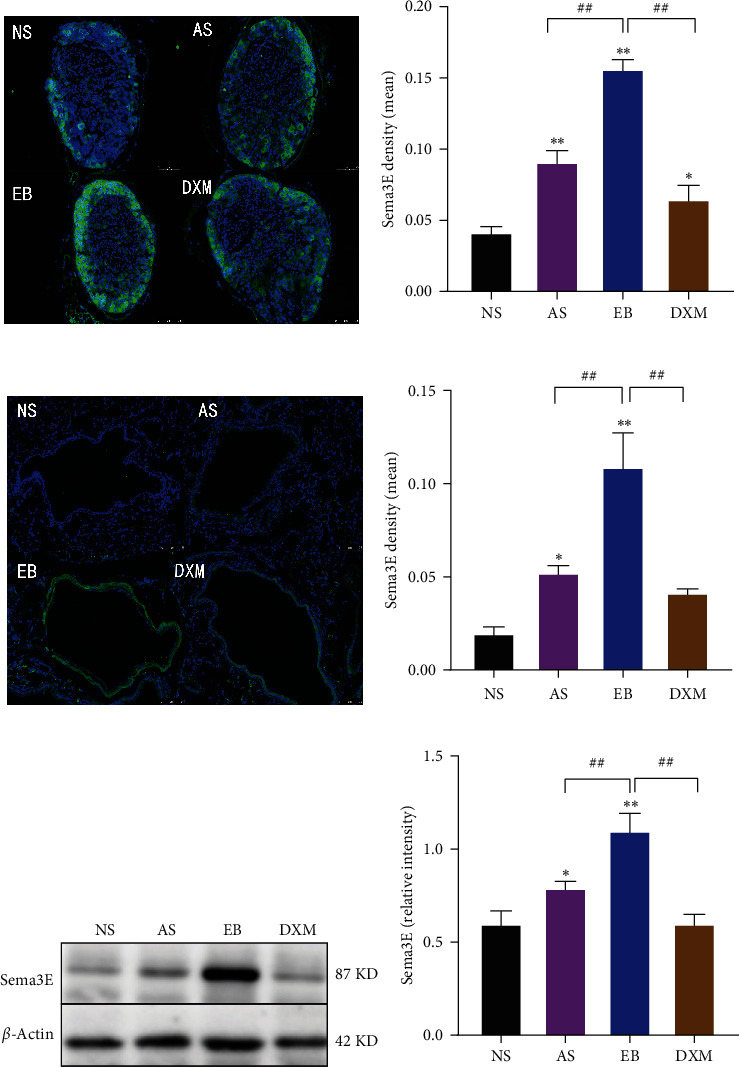
The expression of Sema3E in mouse vagal ganglion and lung tissue. (a) Representative image of Sema3E immunofluorescence staining of mouse vagal ganglion. DNA staining was blue, and Sema3E staining was green original magnifications: ×200, scale bar = 75 *μ*m. (b) The expression level of Sema3E in the mouse vagal ganglion. (c) Representative image of Sema3E immunofluorescence staining of mouse lung tissue. Original magnifications: ×200, scale bar = 75 *μ*m. (d) Sema3E expression level in mouse lung tissue. (e) Western blot analysis of Sema3E levels in lung tissue. (f) Western blot statistical results of Sema3E in mouse lung. (Compared with NS group,  ^*∗*^: *p* < 0.05;  ^*∗∗*^: *p* < 0.01; compared with EB group, ^#^: *p* < 0.05; ^##^: *p* < 0.01). Data were expressed as mean ± standard deviation (*n* = 6). (NS: nasal saline group, AS: asthma group, EB: eosinophilic bronchitis group, DXM: dexamethasone group, Sema3E: Semaphorin3E).

The expression of Sema3E immunofluorescence staining in the vagal ganglion in the AS group, EB group, and DXM group was higher than that in the NS group (*p* < 0.05); Compared with the AS group, the expression of Sema3E in the EB group was increased significantly (*p* < 0.01). Compared with the EB group, the expression of Sema3E was reduced in the DXM group (*p* < 0.01) (Figures 3(a) and 3(b)). Similarly, significant increase in the expression of Sema3E in lung tissue of AS and EB group was observed compared with NS group. Compared with the EB group, the expression of Sema3E in the lung tissue of reduced significantly in AS and DXM groups (*p* < 0.01) (Figures 3(c) and 3(d)). Further, western blot results verified the expression of Sema3E in mouse lung tissue, which was consistent with the immunofluorescence staining results (Figures 3(e) and 3(f)).

### 4.3. Sema3E Decreased Airway Hyperresponsiveness rather than Lung Eosinophilic Inflammation in OVA-Induced Asthma Mouse Model

In order to investigate the direct effects of Sema3E on the airway inflammation and airway reactivity of allergic asthma mouse model, mice were sensitized and challenged with OVA to establish AS mouse model, and mice were exposed intranasally to Sema3E 1 hr before intranasal challenge (Figure 1(a)). Exposure to OVA resulted in signs of airway hyperresponsiveness and pulmonary inflammation (Figure 1(b)–1(e)). Analysis of airway resistance revealed pronounced AHR in OVA-induced asthma mice while Sema3E-treated mice exhibited significantly less AHR (Figure 1(b)). OVA-induced AS mice showed high numbers of inflammatory cells while the total inflammatory cell counts between AS and AS + Sema3E groups were not significant (Figures 1(c) and 1(f)). Challenge with recombinant mouse Sema3E could not decreased lung eosinophilic inflammation compared with that in the AS group (Figure 1(e)). OVA-induced asthma mouse model showed an increase of collagen deposition and mucus production compared with NS and AS + Sema3E groups (Figures 1(d) and 1(g), and 1(h)).

## 5. Discussion

The asthma and EB mouse models were established using the methods as previously described [8]. This study was the first to explore the difference in the expression of Sema3E in vagal ganglia and lung tissue of asthma and EB mice. In this study, eosinophilic inflammation and airway reactivity were significantly increased in AS mouse model, while eosinophilic inflammation and higher cough frequency was also obvious with the effective glucocorticoid therapy but absence of the characteristics of AHR in EB groups, suggesting the successful establishment of AS and EB mouse models. In AS and EB models, the expression of Sema3E in lung tissue was increased compared with NS group. However, it was significantly down-regulated the expression in DXM group when compared with EB group, suggesting that Sema3E is positively correlated with airway eosinophilic inflammation, which may become a new indicator for predicting eosinophil inflammation. Previous studies have shown that the eosinophils could constitutively express Sema4D [17], the expression of which increased in autoimmune diseases such as rheumatoid arthritis, systemic sclerosis as well as eosinophilic rhinosinusitis [18–20]. Sema4A, another member of the Semaphorins family, could promote the activation of eosinophils and mediate occurrence of allergic diseases [21]. As a member of the Semaphorins family, Sema3E may also have the effect of promoting eosinophilic inflammation. However, there is no literature report yet. Unexpectedly, Sema3E-treated AS mouse model demonstrated elevated eosinophil numbers in BALF similar to AS group. However, previous study found that the expression of Sema3E in the airways of AS mice induced by house dust mites was decreased compared with NS group, and exogenous Sema3E could reduce eosinophilic inflammation and serum IgE levels in mice [22]. This is inconsistent with our research results, and we assumed that the main cause of the observed conflicting results might stem from different exposure allergens that impacted the mechanisms of the regulation role of Sema3E in eosinophilic inflammation for the biochemical and immunogenic differences between OVA and HDM. In other words, the composition of HDM extract is complex while OVA is a purified protein, the biochemical and immunogenic differences of which will result in the differing outcomes of allergen exposure and internal mechanism, which prompts further study into the specific mechanism of Sema3E in eosinophilic airway inflammation in response to the different antigens.

Our study showed that the expression of Sema3E in lung tissue of EB mice was significantly increased compared with AS mice, suggesting that Sema3E is not only related to airway eosinophilic inflammation but also AHR. What is more, we found exogenous Sema3E could ameliorate AHR, which suggested Sema3E might be a protective factor in AHR. Movassagh et al. [12] found that downregulation of Sema3E could promote the proliferation and contraction of airway smooth muscle cells, and increase airway collagen deposition and mucus production, eventually leading to increased airway responsiveness in dust mite sensitization of Sema3E-/- allergic asthma mouse model, while after Sema3E treatment, the airway hyperresponsiveness was significantly reduced [12], further elucidating the protective effect of Sema3E in asthma, which might become a new therapeutics for human asthma.

The vagus nerve can send nerve fibers to innervate airway smooth muscle and airway epithelium, mediating bronchial contraction, airway hyperresponsiveness, cough and chest tightness, and plays an important role in the physiopathology of the respiratory system [23]. This study found that the expression of Sema3E in the vagal ganglion of EB mice was significantly higher than that in AS mice, and increased significantly in two mice models compared with the NS group, while the expression of Sema3E was downregulated after DXM treatment, suggesting Sema3E in the vagal ganglion is related to AHR and eosinophilic inflammation. It has been found that the fibers from vagus nerve that projects to the lungs and bronchi can release acetylcholine and contract airway smooth muscle through postsynaptic action, which is an important target for the treatment of asthmatic airway hyperresponsiveness [24]. The use of anti-choline drugs has been confirmed to reduce airway inflammation and AHR in asthmatic mice [25]. The airway hyperresponsiveness was reduced in OVA-induced asthma model with inhibition or loss of TRPV1 expression in vagus sensory neurons, but airway inflammation could not be decreased [26, 27], indicating that the function to regulate airway inflammation and AHR of vagus nerve is complex, which can regulate airway inflammation and AHR alone or at the same time. This study found that Sema3E of the vagal ganglion was upregulated in the AS and EB groups and was significantly higher in the EB group than that in the AS group, suggesting that Sema3E may act as an upstream regulator to regulate airway eosinophilic inflammation and AHR. It is well-known that the vagal ganglion is necessary for the cough reflex. The sensory nerve endings of the airway epithelium produce electrical impulses under various chemical and mechanical stimulis to the vagus nerve, which transmits information to the brain and finally induces cough. EB is one of the important causes of clinical chronic cough. The expression of Sema3E in vagal ganglion in AS and EB mice were significantly increased compared with the NS and DXM groups, indicating that Sema3E is involved in the occurrence of chronic cough in EB. Although Sema3E was significantly higher in the vagal ganglion in the EB group, there was no significant difference in the coughs number between AS and EB mice, further suggesting that cough is related to eosinophilic inflammation, and has nothing to do with AHR.

In fact, there were still some limitations in this study. First, we did not detect the expression of the receptor of Sema3E and other inflammatory factors such as IgE, IL-4, and IL-13 in BALF. Second, we did not used gene knockout mouse for validating the effect of Sema3E on asthma AHR and airway inflammation. Third, this study explored preliminary the role of Sema3E in asthma AHR and airway inflammation, and further research is needed on its specific mechanisms.

In summary, we were the first to investigate the expression of Sema3E in lung tissue and vagal ganglion in OVA-sensitized AS and EB models, and found that Sema3E might act as an upstream regulator in the vagal ganglia to regulate AHR and airway eosinophilic inflammation in allergic asthma, which provided new ideas for the follow-up study of the mechanism of AHR in asthma and chronic cough in EB.

## Data Availability

The datasets used and/or analyzed in the current study are available from the corresponding author on reasonable request.

## Abbreviations

Sema3E: Semaphorin3E
AS: Asthma
EB: Eosinophilic bronchitis
NS: Nasal saline
DXM: Dexamethasone
i.n.: Nasal drops
OVA: Ovalbumin
AHR: Airway hyperresponsiveness
MCh: Methacholine
HE: Hematoxylin and eosin
PAS: Periodic acid–Schiff
BALF: Bronchoalveolar lavage fluid
PBS: Phosphate buffered saline
Eos: Eosinophil
Neu: Neutrophil
Mac: Macrophage
Lym: Lymphocyte.
